# Microscopic Theory of Energy Dissipation and Decoherence in Solid-State Quantum Devices: Need for Nonlocal Scattering Models

**DOI:** 10.3390/e20100726

**Published:** 2018-09-21

**Authors:** Rita Claudia Iotti, Fausto Rossi

**Affiliations:** Department of Applied Science and Technology, Politecnico di Torino, Corso Duca degli Abruzzi 24, 10129 Torino, Italy

**Keywords:** semiconductor nanodevices, quantum transport, density-matrix formalism, Wigner-function simulations, nonlocal dissipation models

## Abstract

Energy dissipation and decoherence in state-of-the-art quantum nanomaterials and related nanodevices are routinely described and simulated via local scattering models, namely relaxation-time and Boltzmann-like schemes. The incorporation of such local scattering approaches within the Wigner-function formalism may lead to anomalous results, such as suppression of intersubband relaxation, incorrect thermalization dynamics, and violation of probability-density positivity. The primary goal of this article is to investigate a recently proposed quantum-mechanical (nonlocal) generalization (*Phys. Rev. B* **2017**, *96*, 115420) of semiclassical (local) scattering models, extending such treatment to carrier–carrier interaction, and focusing in particular on the nonlocal character of Pauli-blocking contributions. In order to concretely show the intrinsic limitations of local scattering models, a few simulated experiments of energy dissipation and decoherence in a prototypical quantum-well semiconductor nanostructure are also presented.

## 1. Introduction

Following the seminal paper by Esaki and Tsu [1], artificially tailored as well as self-assembled solid-state nanostructures form the leading edge of semiconductor science and technology [2]. The design of state-of-the-art optoelectronic nanodevices, in fact, heavily exploits the principles of band-gap engineering [3], achieved by confining charge carriers in spatial regions comparable to their de Broglie wavelengths [4]. This, together with the progressive reduction of the typical time-scales involved, pushes device miniaturization toward limits where, in principle [5], the application of the traditional Boltzmann transport theory [6] becomes questionable, and a comparison with more rigorous quantum-transport approaches [7,8,9,10,11,12,13] is desirable; the latter can be qualitatively subdivided into two main classes. On the one hand, so-called double-time approaches based on the nonequilibrium Green’s function technique [14] have been proposed and widely employed; an introduction to the theory of nonequilibrium Green’s functions with applications to many problems in transport and optics of semiconductors can be found in the books by Haug and Jauho [15], Bonitz [16], and Datta [17]. By employing—and further developing and extending—such nonequilibrium Green’s function formalism, a number of groups have recently proposed efficient quantum-transport treatments for the study of various meso- and nanoscale structures as well as of corresponding micro- and optoelectronic devices [18,19,20,21]. On the other hand, so-called single-time approaches based on the density-matrix formalism [22,23] have been proposed, including phase-space treatments based on the Wigner-function formalism [7,24]. In spite of the intrinsic validity limits of the semiclassical theory just recalled, during the last few decades, a number of Boltzmann-like Monte Carlo simulation schemes have been successfully employed for the investigation of new-generation semiconductor nanodevices [25,26,27,28,29,30,31,32,33,34,35,36]. Such modeling strategies—based on the neglect of carrier phase coherence—are, however, unable to properly describe ultrafast phenomena. To this aim, the crucial step is to adopt a quantum-mechanical description of the carrier subsystem; this can be performed at different levels, ranging from phenomenological dissipation and decoherence models [37] to quantum-kinetic treatments [8,10,11]. Indeed, in order to overcome the intrinsic limitations of the semiclassical picture in properly describing ultrafast space-dependent phenomena —e.g., real-space transfer and escape versus capture processes— Jacoboni and co-workers have proposed a quantum Monte Carlo technique [38], while Kuhn and co-workers have proposed a quantum-kinetic treatment [39]; however, due to their high computational cost, these non-Markovian density-matrix approaches are often unsuitable for the design and optimization of new-generation nanodevices.

In order to overcome such limitations, a conceptually simple as well as physically reliable quantum-mechanical generalization of the conventional Boltzmann theory has been recently proposed [40]. The latter, based on the density-matrix formalism, preserves the power and flexibility of the semiclassical picture in describing a large variety of scattering mechanisms; more specifically, employing a microscopic derivation of generalized scattering rates based on a reformulation of the Markov limit [41], a density-matrix equation has been derived, able to properly account for space-dependent ultrafast dynamics in semiconductor nanostructures. Indeed, the density-matrix approach just recalled has been recently applied to the investigation of scattering nonlocality in GaN-based materials [42] and carbon nanotubes [43], as well as to the study of carrier capture processes [44]. It is worth mentioning that a purely phenomenological Lindblad-type approach [45] based on the jump-operator formalism has been recently proposed [46].

In addition to the density-matrix treatments just recalled, quantum-transport phenomena have been extensively investigated via Wigner-function approaches [7,47]. Indeed, the Wigner-function formalism has been adopted in various contexts to study ultrashort space- and/or time-scale phenomena in semiconductor nanomaterials and related nanodevices [48,49,50,51,52,53,54,55,56,57,58,59,60,61,62,63,64,65,66,67,68,69,70,71,72,73,74,75,76,77,78]. In view of their formal similarity with the conventional Boltzmann theory, in these Wigner-function treatments, dissipation versus decoherence phenomena are often accounted for in semiclassical terms via local scattering models, such as relaxation-time and Boltzmann-like schemes. It has been recently shown [79] that the use of such local scattering approaches may lead to unphysical results, namely anomalous suppression of intersubband relaxation, incorrect thermalization dynamics, and violation of probability-density positivity. To overcome such severe limitations, in [79], a quantum-mechanical generalization of relaxation-time and Boltzmann-like models has recently been proposed, resulting in nonlocal electron-phonon scattering superoperators.

The goal of this paper is twofold: on the one hand, we shall elucidate the intimate link between density-matrix and Wigner-function approaches, pointing out intrinsic limitations of semiclassical scattering models within these, apparently different, simulation strategies. On the other hand, we shall extend the carrier–phonon treatment in [79] to carrier–carrier interaction; indeed, the latter has been for a long time to have a dramatic impact both on optical properties [8,10,11] as well as on transport phenomena [80,81], and has more recently been in the spotlight due to the effects of its interplay with spin-orbit coupling [82,83,84,85]. Moreover, we shall investigate in more detail the role played by Pauli-blocking terms both within the density matrix formalism (population versus polarization contributions) as well as within the Wigner-function picture (local versus nonlocal action). In order to concretely show the intrinsic limitations of local scattering models, a few simulated experiments of energy dissipation and decoherence in a prototypical quantum-well semiconductor nanostructure are also presented.

The paper is organized as follows: in Section 2, we shall briefly recall the main features of semiclassical scattering models, both for bulk and for nanostructured materials. In Section 3, we shall provide a fully quantum-mechanical treatment of energy-dissipation and decoherence phenomena within the density-matrix formalism, and we shall translate the latter into a nonlocal Wigner-function scattering model for both carrier–phonon and carrier–carrier interaction. In Section 4, we shall analyze the role played by Pauli-blocking contributions, discussing non-classical features, like polarization scattering within the density-matrix formalism, and nonlocal Pauli factors within the Wigner-function picture. Finally, in Section 5, we shall summarize and draw a few conclusions.

## 2. Semiclassical Scattering Models

To investigate in quantum-mechanical terms the electro-optical response of semiconductor nanomaterials and related nanodevices, it is crucial to study the time evolution of single-particle quantities, e.g., total carrier density, mean kinetic energy, charge current, etc. Such quantities may be conveniently expressed by a suitable (quantum-plus-statistical) average of a corresponding (single-particle) operator in terms of the single-particle density matrix $\rho_{\alpha_{1}\alpha_{2}}$ [23] (α denoting the electronic single-particle states of our nanostructure): its diagonal terms $f_{\alpha }=\rho_{\alpha \alpha }$ describe the population of the generic single-particle state α while the off-diagonal terms describe the quantum-mechanical phase coherence (or polarization) between states $\alpha_{1}$ and $\alpha_{2}$. More precisely, we may write:

(1) $$\rho_{\alpha_{1}\alpha_{2}}=f_{\alpha_{1}}\delta_{\alpha_{1}\alpha_{2}}+p_{\alpha_{1}\alpha_{2}}.\;$$

Here, the first (diagonal) term describes the semiclassical state populations, while the second term

(2) $$p_{\alpha_{1}\alpha_{2}}=\rho_{\alpha_{1}\alpha_{2}}\left(1-\delta_{\alpha_{1}\alpha_{2}}\right)$$

is the so-called polarization matrix.

Regardless of the specific physical system and related modelling, the time evolution of the single-particle density matrix can be always expressed as the sum of a deterministic (d) and of a scattering (s) contribution:

(3) $$\frac{\partial \rho_{\alpha_{1}\alpha_{2}}}{\partial t}={\left.\frac{\partial \rho_{\alpha_{1}\alpha_{2}}}{\partial t}\right|}_{d}+{\left.\frac{\partial \rho_{\alpha_{1}\alpha_{2}}}{\partial t}\right|}_{s}.\;$$

Here,

(4) $${\left.\frac{\partial \rho_{\alpha_{1}\alpha_{2}}}{\partial t}\right|}_{d}=\frac{\epsilon_{\alpha_{1}}-\epsilon_{\alpha_{2}}}{ı\hbar }\;\rho_{\alpha_{1}\alpha_{2}}$$

($\epsilon_{\alpha }$ denoting the energy of the single-particle state α), while the explicit form of the scattering contribution depends on our level of description (see Section 3).

As discussed in detail in [13], for quantum nanodevices characterized by a relevant dissipation versus decoherence dynamics and operating in steady-state conditions, it is common practice to adopt the so-called semiclassical picture; this amounts to neglecting the polarization term in (2). Within such semiclassical (or diagonal) approximation ($\rho_{\alpha_{1}\alpha_{2}}=f_{\alpha_{1}}\delta_{\alpha_{1}\alpha_{2}}$), the simplest scattering model is given by the well-known relaxation-time approximation (RTA) [23]:

(5) $${\left.\frac{\partial f_{\alpha }}{\partial t}\right|}_{s}=-\Gamma_{\alpha }\;\left(f_{\alpha }^{}-f_{\alpha }^{\circ }\right).\;$$

Here, the relaxation of the state population $f_{\alpha }$ toward the equilibrium population $f_{\alpha }^{\circ }$ is described in terms of a state-dependent relaxation rate $\Gamma_{\alpha }$ that purely depends on that state and encodes all relevant scattering processes characterizing the operational conditions of the device.

In order to provide a more accurate description of nonequilibrium phenomena, the RTA model in Equation (5) is usually replaced by a Boltzmann-like scattering model of the form:

(6) $${\left.\frac{\partial f_{\alpha }}{\partial t}\right|}_{s}=\sum_{s}\sum_{\alpha^{\prime }}\left(\left(1-f_{\alpha }\right)P_{\alpha \alpha^{\prime }}^{s}f_{\alpha^{\prime }}-\left(1-f_{\alpha^{\prime }}\right)P_{\alpha^{\prime }\alpha }^{s}f_{\alpha }\right).\;$$

The above collision term exhibits the well-known in- minus out-scattering structure, and allows one to incorporate a number of scattering mechanisms *s* via corresponding scattering rates $P_{\alpha^{\prime }\alpha }^{s}$; the latter describes the probability per time unit for an electronic transition $\alpha \to \alpha^{\prime }$ induced by the scattering mechanism *s*, and are typically derived via the standard Fermi’s golden rule; moreover, here the factors $\left(1-f_{\alpha }\right)$ describe Pauli-blocking effects (see below).

As anticipated in the introductory section, in addition to the density-matrix treatments just recalled, state-of-the-art quantum nanodevices are often modelled via Wigner-function-based simulation schemes [48,49,50,51,52,53,54,55,56,57,58,59,60,61,62,63,64,65,66,67,68,69,70,71,72,73,74,75,76,77,78]. Regardless of the specific problem under investigation, the time evolution of the single-particle Wigner function f(r,k) can be expressed once again as the sum of a deterministic and of a scattering contribution, namely [86]:

(7) $$\frac{\partial f(r,k)}{\partial t}={\left.\frac{\partial f(r,k)}{\partial t}\right|}_{d}+{\left.\frac{\partial f(r,k)}{\partial t}\right|}_{s}.$$

Here, the first term is the quantum-mechanical generalization of the deterministic (diffusion-plus-drift) term in the semiclassical theory, and can be conveniently expressed in terms of the well-known Moyal brackets [87], whose explicit form depends on the electron band dispersion and on the electromagnetic gauge [72,79]. The second term, in contrast, describes again energy dissipation and decoherence phenomena induced by various scattering mechanisms. Within a fully quantum-mechanical treatment, such a scattering term is strictly nonlocal, as described in detail in [42], and is of the general form

(8) $${\left.\frac{\partial f(r,k)}{\partial t}\right|}_{s}=S\left[f(r^{\prime },k^{\prime })\right]\left(r,k\right)\;,$$

where, in general, *S* is a nonlinear scattering superoperator describing a nonlocal action both in r and k, i.e., the scattering contribution to the generic phase-space point (r,k) depends on the value of the Wigner function *f* in any other phase-space point $\left(r^{\prime },k^{\prime }\right)$.

Due to the difficulty in dealing with its fully nonlocal character, it is common practice in many quantum-simulation approaches to replace the scattering superoperator in Equation (8) with a local superoperator. The simplest choice is once again the adoption of an RTA model [49,51,66,75] that rewords the semiclassical case, namely:

(9) $${\left.\frac{\partial f(r,k)}{\partial t}\right|}_{s}=-\Gamma \left(r,k\right)\;\left(f\left(r,k\right)-f^{\circ }\left(r,k\right)\right).\;$$

Here, similar to the RTA model in (5), the relaxation of the Wigner function in the phase-space point (r,k) toward the equilibrium Wigner function $f^{\circ }\left(r,k\right)$ is described in terms of a space- and momentum-dependent relaxation rate Γ(r,k); the latter may be extracted from fully microscopic Monte Carlo simulations [6], or modelled via simplified Fermi’s Golden-rule treatments.

Another simplified (i.e., local) version of the scattering superoperator in Equation (8) is inspired again by the formal analogy between the Wigner transport equation in (7) and the usual Boltzmann transport theory, and consists of replacing *S* with a conventional (i.e., semiclassical) Boltzmann collision term [6,23]:

(10) $${\left.\frac{\partial f(r,k)}{\partial t}\right|}_{s}=\sum_{s}\int dk^{\prime }\left[P^{s}\left(r;k,k^{\prime }\right)f\left(r,k^{\prime }\right)-P^{s}\left(r;k^{\prime },k\right)f\left(r,k\right)\right],$$

where

(11) $$P^{s}\left(r;k,k^{\prime }\right)=\left(1-f(r,k)\right)P_{0}^{s}\left(r;k,k^{\prime }\right)$$

denotes the low-density scattering rate $P_{0}^{s}$ in r (for the generic transition $k^{\prime }\to k$ induced by the scattering mechanism *s*) weighted by the usual Pauli-blocking factor, and simply reduces to $P_{0}^{s}\left(r;k,k^{\prime }\right)$ in the low-density limit (f(r,k)→0).

The Boltzmann collision term in (10) is characterized once again by the well-established in- minus out-scattering structure; indeed, the latter may also be written as

(12) $$\begin{array}{ccc} {\left.\frac{\partial f(r,k)}{\partial t}\right|}_{s} & = & \sum_{s}\int dr^{\prime }\;dk^{\prime }P^{s,\mathrm{in}}\left(r,k;r^{\prime },k^{\prime }\right)f\left(r^{\prime },k^{\prime }\right)\\ & - & \sum_{s}\int dr^{\prime }\;dk^{\prime }P^{s,\mathrm{out}}\left(r,k;r^{\prime },k^{\prime }\right)f\left(r^{\prime },k^{\prime }\right) \end{array}$$

with

(13) $$P^{s,\mathrm{in}}\left(r,k;r^{\prime },k^{\prime }\right)=\delta \left(r-r^{\prime }\right)P^{s}\left(r;k,k^{\prime }\right)$$

and

(14) $$P^{s,\mathrm{out}}\left(r,k;r^{\prime },k^{\prime }\right)=\delta \left(r-r^{\prime }\right)\delta \left(k-k^{\prime }\right)\int dk^{\prime \prime }P^{s}\left(r;k^{\prime \prime },k\right)\;,$$

which shows that, within the conventional Boltzmann theory, both superoperators are local in r, and that the out-scattering one is local in k as well.

## 3. Fully Quantum-Mechanical Scattering Models

The quantum-mechanical derivation of effective scattering models within the density-matrix formalism may involve one or more of the following three key steps [88]: (i) mean-field approximation; (ii) adiabatic or Markov limit; and (iii) semiclassical or diagonal limit.

When all of these three approximations are applied, the usual Boltzmann collision term is obtained (see Equation (6)); the latter, if applicable (see above), constitutes a robust/reliable particle-like description in purely stochastic terms, thus providing physically acceptable results.

In contrast, the combination of the first two approximation schemes only, namely mean-field treatment and adiabatic limit, allows one to derive so-called Markovian scattering superoperators, whose action may lead to unphysical results [89]. Indeed, as originally pointed out by Spohn and coworkers [90], the choice of the adiabatic decoupling strategy is definitely not unique and, in general, the positive-definite character of the density-matrix operator may be violated.

To overcome this severe limitation, a few years ago, an alternative and more general Markov procedure has been proposed [41]; the latter allows for a microscopic derivation of Lindblad-type scattering superoperators [45], thus preserving the positive-definite nature of the electronic quantum-mechanical state. More recently [40], such alternative Markov scheme combined with the conventional mean-field approximation just recalled has allowed for the derivation of positive-definite nonlinear scattering superoperators acting on the single-particle density matrix $\rho_{\alpha_{1}\alpha_{2}}$; more specifically, as shown in [40], for both carrier–phonon and carrier–carrier interaction, the resulting single-particle equation is given by

(15) $${\left.\frac{d\rho_{\alpha_{1}\alpha_{2}}}{dt}\right|}_{s}=\frac{1}{2}\sum_{\alpha^{\prime }\alpha_{1}^{\prime }\alpha_{2}^{\prime }}\left(\left(\delta_{\alpha_{1}\alpha^{\prime }}-\rho_{\alpha_{1}\alpha^{\prime }}\right)P_{\alpha^{\prime }\alpha_{2},\alpha_{1}^{\prime }\alpha_{2}^{\prime }}^{s}\rho_{\alpha_{1}^{\prime }\alpha_{2}^{\prime }}-\left(\delta_{\alpha^{\prime }\alpha_{1}^{\prime }}-\rho_{\alpha^{\prime }\alpha_{1}^{\prime }}\right)P_{\alpha^{\prime }\alpha_{1}^{\prime },\alpha_{1}\alpha_{2}^{\prime }}^{s*}\rho_{\alpha_{2}^{\prime }\alpha_{2}}\right)\;+\;H.c.$$

with generalized carrier–phonon scattering rates [91]

(16) $$P_{\alpha_{1}\alpha_{2},\alpha_{1}^{\prime }\alpha_{2}^{\prime }}^{\mathrm{cp}}=A_{\alpha_{1}\alpha_{1}^{\prime }}^{\mathrm{cp}}A_{\alpha_{2}\alpha_{2}^{\prime }}^{\mathrm{cp}*}$$

and generalized carrier–carrier scattering rates [92]

(17) $$P_{\alpha_{1}\alpha_{2},\alpha_{1}^{\prime }\alpha_{2}^{\prime }}^{\mathrm{cc}}=2\sum_{{\bar{\alpha }}_{1}{\bar{\alpha }}_{2},{\bar{\alpha }}_{1}^{\prime }{\bar{\alpha }}_{2}^{\prime }}\left(\delta_{{\bar{\alpha }}_{2}{\bar{\alpha }}_{1}}-\rho_{{\bar{\alpha }}_{2}{\bar{\alpha }}_{1}}\right)A_{\alpha_{1}{\bar{\alpha }}_{1},\alpha_{1}^{\prime }{\bar{\alpha }}_{1}^{\prime }}^{\mathrm{cc}}A_{\alpha_{2}{\bar{\alpha }}_{2},\alpha_{2}^{\prime }{\bar{\alpha }}_{2}^{\prime }}^{\mathrm{cc}*}\rho_{{\bar{\alpha }}_{1}^{\prime }{\bar{\alpha }}_{2}^{\prime }}\;.$$

Here, $A_{\alpha \alpha^{\prime }}^{\mathrm{cp}}$ denotes the matrix element of the corresponding carrier–phonon Lindblad operator for the (one-body) transition $\alpha^{\prime }\to \alpha$, while $A_{\alpha \bar{\alpha },\alpha^{\prime }{\bar{\alpha }}^{\prime }}^{\mathrm{cc}}$ denotes the matrix element of the corresponding carrier–carrier Lindblad operator for the (two-body) transition $\alpha^{\prime }{\bar{\alpha }}^{\prime }\to \alpha \bar{\alpha }$. These carrier–phonon and carrier–carrier Lindblad matrix elements can be microscopically derived starting from the corresponding interaction Hamiltonians, as described in [40].

It is worth stressing that, contrary to the generalized carrier–phonon rates in (16), the generalized carrier–carrier rates in (17) are themselves a function of the single-particle density matrix; this is a clear fingerprint of the two-body nature of the carrier–carrier interaction (see below).

The generic single-particle scattering superoperator in (15) is the result of positive-like (in-scattering) and negative-like (out-scattering) contributions, which are nonlinear functions of the single-particle density matrix. Indeed, in the semiclassical limit previously recalled ($\rho_{\alpha_{1}\alpha_{2}}=f_{\alpha_{1}}\delta_{\alpha_{1}\alpha_{2}}$), the density-matrix Equation (15) assumes the expected nonlinear Boltzmann-type form

(18) $${\left.\frac{df_{\alpha }}{dt}\right|}_{s}=\sum_{\alpha^{\prime }}\left(\left(1-f_{\alpha }\right)P_{\alpha \alpha^{\prime }}^{s}f_{\alpha^{\prime }}-\left(1-f_{\alpha^{\prime }}\right)P_{\alpha^{\prime }\alpha }^{s}f_{\alpha }\right)$$

with semiclassical carrier–phonon scattering rates

(19) $$P_{\alpha \alpha^{\prime }}^{\mathrm{cp}}=P_{\alpha \alpha ,\alpha^{\prime }\alpha^{\prime }}^{\mathrm{cp}}={\left|A_{\alpha \alpha^{\prime }}^{\mathrm{cp}}\right|}^{2}$$

and semiclassical carrier–carrier scattering rates

(20) $$P_{\alpha \alpha^{\prime }}^{\mathrm{cc}}=P_{\alpha \alpha ,\alpha^{\prime }\alpha^{\prime }}^{\mathrm{cc}}=2\sum_{\bar{\alpha }{\bar{\alpha }}^{\prime }}\left(1-f_{\bar{\alpha }}\right){\left|A_{\alpha \bar{\alpha },\alpha^{\prime }{\bar{\alpha }}^{\prime }}^{\mathrm{cc}}\right|}^{2}f_{{\bar{\alpha }}^{\prime }}.\;$$

The above semiclassical limit clearly shows that the nonlinearity factors $\left(\delta_{\alpha_{1}\alpha_{2}}-\rho_{\alpha_{1}\alpha_{2}}\right)$ in (15) as well as in (17) can be regarded as the quantum-mechanical generalization of the Pauli factors $\left(1-f_{\alpha }\right)$ of the conventional Boltzmann theory (see also Section 4 below).

A closer inspection of Equations (15) and (17)—together with their semiclassical counterparts in (18) and (20)—confirms the two-body nature of the carrier–carrier interaction. Indeed, differently from the carrier–phonon scattering, in this case, the density-matrix equation describes the time evolution of a so-called “main carrier” α interacting with a so-called “partner carrier” $\bar{\alpha }$.

As already pointed out in the introductory section, in addition to the density-matrix treatments just recalled, quantum-transport phenomena in nanomaterials and related nanodevices have been extensively investigated via Wigner-function approaches [48,49,50,51,52,53,54,55,56,57,58,59,60,61,62,63,64,65,66,67,68,69,70,71,72,73,74,75,76,77,78]. In view of their formal similarity with the conventional Boltzmann transport theory, in these Wigner-function treatments, dissipation versus decoherence phenomena are often accounted for via local scattering models, such as relaxation-time and Boltzmann-like schemes (see Section 2).

In spite of the fact that density-matrix and Wigner-function treatments have been historically developed and applied independently to the modeling and optimization of various state-of-the-art nanodevices, it is imperative to stress that the single-particle density matrix $\rho_{\alpha_{1}\alpha_{2}}$ in (3) is linked to the single-particle Wigner function f(r,k) in (7) via a one-to-one correspondence provided by the well-known Weyl–Wigner transform [7]. More specifically, adopting the very same notation employed in [72], we have

(21) $$f\left(r,k\right)=\sum_{\alpha_{1}\alpha_{2}}W_{\alpha_{1}\alpha_{2}}^{*}\left(r,k\right)\rho_{\alpha_{1}\alpha_{2}}\;,$$

where

(22) $$W_{\alpha_{1}\alpha_{2}}^{}\left(r,k\right)=\int dr^{\prime }\phi_{\alpha_{1}}^{}\left(r+\frac{r^{\prime }}{2}\right)e^{-ık\cdot r^{\prime }}\phi_{\alpha_{2}}^{*}\left(r-\frac{r^{\prime }}{2}\right)$$

denotes the Weyl–Wigner transform just recalled, and $\phi_{\alpha }\left(r\right)$ the real-space wavefunction of the single-particle state α.

In view of such one-to-one correspondence, it is thus clear that, given a scattering model within the density-matrix picture, the latter will have a well-defined Wigner-function counterpart, and vice versa. On this basis, the most natural and rigorous approach is to select a reliable/robust model in one picture, and then to translate it into the other one via the Weyl–Wigner transform in (22). This is exactly what has been recently proposed in [79]: applying the nonlinear density-matrix scattering model in (15) to the case of carrier–phonon interaction, a nonlocal scattering superoperator for the Wigner function has been derived. In what follows, we shall extend such nonlocal scattering treatment to the case of carrier–carrier interaction as well.

In order to get the desired Wigner-function version of the density-matrix scattering superoperator in (15), the crucial step is to apply to the latter the Weyl–Wigner transform (21) together with its inverse, namely [93]

(23) $$\rho_{\alpha_{1}\alpha_{2}}=\int \frac{dr\;dk}{{\left(2\pi \right)}^{3}}W_{\alpha_{1}\alpha_{2}}^{}\left(r,k\right)f\left(r,k\right).\;$$

The resulting Wigner-function scattering superoperator is given by

(24) $$\begin{array}{ccc} {\left.\frac{\partial f(r,k)}{\partial t}\right|}_{s} & = & \int dr^{\prime }\;dk^{\prime }P^{s,\mathrm{in}}\left(r,k;r^{\prime },k^{\prime }\right)f\left(r^{\prime },k^{\prime }\right)\\ & - & \int dr^{\prime }\;dk^{\prime }P^{s,\mathrm{out}}\left(r,k;r^{\prime },k^{\prime }\right)f\left(r^{\prime },k^{\prime }\right), \end{array}$$

where

(25) $$P^{s,\mathrm{in}/\mathrm{out}}\left(r,k;r^{\prime },k^{\prime }\right)=\int \frac{dr^{\prime \prime }\;dk^{\prime \prime }}{{\left(2\pi \right)}^{3}}\;\left(1-f(r^{\prime \prime },k^{\prime \prime })\right){\tilde{P}}^{s,\mathrm{in}/\mathrm{out}}\left(r^{\prime \prime },k^{\prime \prime };r,k;r^{\prime },k^{\prime }\right)$$

with

(26) $${\tilde{P}}^{s,\mathrm{in}}\left(r^{\prime \prime },k^{\prime \prime };r,k;r^{\prime },k^{\prime }\right)=\frac{1}{{\left(2\pi \right)}^{3}}\sum_{\alpha_{1}\alpha_{2}\alpha^{\prime }\alpha_{1}^{\prime }\alpha_{2}^{\prime }}ℜ\left\{W_{\alpha_{1}\alpha_{2}}^{}\left(r,k\right)W_{\alpha_{1}\alpha^{\prime }}^{*}\left(r^{\prime \prime },k^{\prime \prime }\right)P_{\alpha^{\prime }\alpha_{2},\alpha_{1}^{\prime }\alpha_{2}^{\prime }}^{s}W_{\alpha_{1}^{\prime }\alpha_{2}^{\prime }}^{*}\left(r^{\prime },k^{\prime }\right)\right\}$$

and

(27) $${\tilde{P}}^{s,\mathrm{out}}\left(r^{\prime \prime },k^{\prime \prime };r,k;r^{\prime },k^{\prime }\right)=\frac{1}{{\left(2\pi \right)}^{3}}\sum_{\alpha_{1}\alpha_{2}\alpha^{\prime }\alpha_{1}^{\prime }\alpha_{2}^{\prime }}ℜ\left\{W_{\alpha_{1}\alpha_{2}}^{}\left(r,k\right)W_{\alpha^{\prime }\alpha_{1}^{\prime }}^{*}\left(r^{\prime \prime },k^{\prime \prime }\right)P_{\alpha^{\prime }\alpha_{1}^{\prime },\alpha_{1}\alpha_{2}^{\prime }}^{s\;*}W_{\alpha_{2}^{\prime }\alpha_{2}}^{*}\left(r^{\prime },k^{\prime }\right)\right\}\;.$$

As expected, for both carrier–phonon and carrier–carrier interaction, the proposed quantum-mechanical generalization of the standard Boltzmann collision term in (10) is thus intrinsically nonlocal. In particular, comparing Equation (25) with its semiclassical counterpart in (11), we realize that the action of the Pauli exclusion principle within the Wigner phase-space is itself nonlocal: the generalized in and out scattering rates for a given transition $r,k\to r^{\prime },k^{\prime }$ depend on the value of the Wigner function in any other phase-space point $r^{\prime \prime },k^{\prime \prime }$ via the Pauli factor $1-f(r^{\prime \prime },k^{\prime \prime })$. Such Pauli-blocking nonlocality will be discussed in more detail at the end of Section 4.

In the low-density limit (f(r,k)→0), the proposed scattering model in (25) reduces to:

(28) $$P^{s,\mathrm{in}}\left(r,k;r^{\prime },k^{\prime }\right)=\frac{1}{{\left(2\pi \right)}^{3}}\sum_{\alpha_{1}\alpha_{2}\alpha_{1}^{\prime }\alpha_{2}^{\prime }}ℜ\left\{W_{\alpha_{1}\alpha_{2}}^{}\left(r,k\right)P_{\alpha_{1}\alpha_{2},\alpha_{1}^{\prime }\alpha_{2}^{\prime }}^{s}W_{\alpha_{1}^{\prime }\alpha_{2}^{\prime }}^{*}\left(r^{\prime },k^{\prime }\right)\right\}$$

and

(29) $$P^{s,\mathrm{out}}\left(r,k;r^{\prime },k^{\prime }\right)=\frac{1}{{\left(2\pi \right)}^{3}}\sum_{\alpha_{1}\alpha_{2}\alpha_{1}^{\prime }\alpha_{2}^{\prime }}ℜ\left\{W_{\alpha_{1}\alpha_{2}}^{}\left(r,k\right)P_{\alpha_{1}^{\prime }\alpha_{1}^{\prime },\alpha_{1}\alpha_{2}^{\prime }}^{s\;*}W_{\alpha_{2}^{\prime }\alpha_{2}}^{*}\left(r^{\prime },k^{\prime }\right)\right\}\;.$$

It is however worth stressing that, while for carrier–phonon interaction the above low-density scattering rates are different from zero, for carrier–carrier interaction, the latter vanish; this is due to the fact that, in the low-density limit ($\rho_{\alpha_{1}\alpha_{2}}\to 0$), the generalized carrier–carrier scattering rates in (17) tend to zero.

For the case of carrier–phonon interaction, we may easily derive the explicit form of the corresponding Wigner-function scattering rates. By inserting Equation (16) into Equations (26) and (27), we get

(30) $${\tilde{P}}^{\mathrm{cp},\mathrm{in}}\left(r^{\prime \prime },k^{\prime \prime };r,k;r^{\prime },k^{\prime }\right)=\frac{1}{{\left(2\pi \right)}^{3}}\sum_{\alpha_{1}\alpha_{2}\alpha^{\prime }\alpha_{1}^{\prime }\alpha_{2}^{\prime }}ℜ\left\{W_{\alpha_{1}\alpha_{2}}^{}\left(r,k\right)W_{\alpha_{1}\alpha^{\prime }}^{*}\left(r^{\prime \prime },k^{\prime \prime }\right)A_{\alpha^{\prime }\alpha_{1}^{\prime }}^{\mathrm{cp}}A_{\alpha_{2}\alpha_{2}^{\prime }}^{\mathrm{cp}*}W_{\alpha_{1}^{\prime }\alpha_{2}^{\prime }}^{*}\left(r^{\prime },k^{\prime }\right)\right\}$$

and

(31) $${\tilde{P}}^{\mathrm{cp},\mathrm{out}}\left(r^{\prime \prime },k^{\prime \prime };r,k;r^{\prime },k^{\prime }\right)=\frac{1}{{\left(2\pi \right)}^{3}}\sum_{\alpha_{1}\alpha_{2}\alpha^{\prime }\alpha_{1}^{\prime }\alpha_{2}^{\prime }}ℜ\left\{W_{\alpha_{1}\alpha_{2}}^{}\left(r,k\right)W_{\alpha^{\prime }\alpha_{1}^{\prime }}^{*}\left(r^{\prime \prime },k^{\prime \prime }\right)A_{\alpha^{\prime }\alpha_{1}}^{\mathrm{cp}*}A_{\alpha_{1}^{\prime }\alpha_{2}^{\prime }}^{\mathrm{cp}}W_{\alpha_{2}^{\prime }\alpha_{2}}^{*}\left(r^{\prime },k^{\prime }\right)\right\}\;.$$

In contrast, for the case of carrier–carrier interaction, getting the explicit form of the corresponding Wigner-function scattering rates is not so straightforward. To this aim, the first step is to rewrite the generalized carrier–carrier rates in (17) in terms of the Wigner function f(r,k). More specifically, by inserting into Equation (17) the inverse Weyl–Wigner transform (23), we get:

$$P_{\alpha_{1}\alpha_{2},\alpha_{1}^{\prime }\alpha_{2}^{\prime }}^{\mathrm{cc}}=2\sum_{{\bar{\alpha }}_{1}{\bar{\alpha }}_{2},{\bar{\alpha }}_{1}^{\prime }{\bar{\alpha }}_{2}^{\prime }}\int \frac{d{\bar{r}}^{\prime \prime }d{\bar{k}}^{\prime \prime }\;d{\bar{r}}^{\prime }d{\bar{k}}^{\prime }}{{\left(2\pi \right)}^{6}}\;\cdot$$

(32) $$\left(1-f({\bar{r}}^{\prime \prime },{\bar{k}}^{\prime \prime })\right)W_{{\bar{\alpha }}_{2}{\bar{\alpha }}_{1}}^{}\left({\bar{r}}^{\prime \prime },{\bar{k}}^{\prime \prime }\right)A_{\alpha_{1}{\bar{\alpha }}_{1},\alpha_{1}^{\prime }{\bar{\alpha }}_{1}^{\prime }}^{\mathrm{cc}}A_{\alpha_{2}{\bar{\alpha }}_{2},\alpha_{2}^{\prime }{\bar{\alpha }}_{2}^{\prime }}^{\mathrm{cc}*}W_{{\bar{\alpha }}_{1}^{\prime }{\bar{\alpha }}_{2}^{\prime }}^{}\left({\bar{r}}^{\prime },{\bar{k}}^{\prime }\right)f\left({\bar{r}}^{\prime },{\bar{k}}^{\prime }\right).$$

By inserting this last expression into Equations (26) and (27), the latter can be compactly rewritten as

(33) $${\tilde{P}}^{\mathrm{cc},\mathrm{in}/\mathrm{out}}\left(r^{\prime \prime }\;,\;k^{\prime \prime };r\;,\;k;r^{\prime }\;,\;k^{\prime }\right)\;\;=\;\;\int \frac{d{\bar{r}}^{\prime \prime }d{\bar{k}}^{\prime \prime }\;d{\bar{r}}^{\prime }d{\bar{k}}^{\prime }}{{\left(2\pi \right)}^{6}}\left(1\;-\;f({\bar{r}}^{\prime \prime }\;,\;{\bar{k}}^{\prime \prime })\right){\tilde{p}}^{\mathrm{in}/\mathrm{out}}\left({\bar{r}}^{\prime \prime }\;,\;{\bar{k}}^{\prime \prime };r^{\prime \prime }\;,\;k^{\prime \prime };r\;,\;k;r^{\prime }\;,\;k^{\prime };{\bar{r}}^{\prime }\;,\;{\bar{k}}^{\prime }\right)f\left({\bar{r}}^{\prime }\;,\;{\bar{k}}^{\prime }\right)$$

with

$${\tilde{p}}^{\mathrm{in}}\left({\bar{r}}^{\prime \prime },{\bar{k}}^{\prime \prime };r^{\prime \prime },k^{\prime \prime };r,k;r^{\prime },k^{\prime };{\bar{r}}^{\prime },{\bar{k}}^{\prime }\right)=\frac{1}{4\pi^{3}}\sum_{\alpha_{1}\alpha_{2}\alpha^{\prime }\alpha_{1}^{\prime }\alpha_{2}^{\prime }}\sum_{{\bar{\alpha }}_{1}{\bar{\alpha }}_{2},{\bar{\alpha }}_{1}^{\prime }{\bar{\alpha }}_{2}^{\prime }}\;\cdot$$

(34) $$ℜ\left\{W_{{\bar{\alpha }}_{2}{\bar{\alpha }}_{1}}^{}\left({\bar{r}}^{\prime \prime },{\bar{k}}^{\prime \prime }\right)W_{\alpha_{1}\alpha^{\prime }}^{*}\left(r^{\prime \prime },k^{\prime \prime }\right)A_{\alpha^{\prime }{\bar{\alpha }}_{1},\alpha_{1}^{\prime }{\bar{\alpha }}_{1}^{\prime }}^{\mathrm{cc}}W_{\alpha_{1}\alpha_{2}}^{}\left(r,k\right)A_{\alpha_{2}{\bar{\alpha }}_{2},\alpha_{2}^{\prime }{\bar{\alpha }}_{2}^{\prime }}^{\mathrm{cc}*}W_{\alpha_{1}^{\prime }\alpha_{2}^{\prime }}^{*}\left(r^{\prime },k^{\prime }\right)W_{{\bar{\alpha }}_{1}^{\prime }{\bar{\alpha }}_{2}^{\prime }}^{}\left({\bar{r}}^{\prime },{\bar{k}}^{\prime }\right)\right\}$$

and

$${\tilde{p}}^{\mathrm{out}}\left({\bar{r}}^{\prime \prime },{\bar{k}}^{\prime \prime };r^{\prime \prime },k^{\prime \prime };r,k;r^{\prime },k^{\prime };{\bar{r}}^{\prime },{\bar{k}}^{\prime }\right)=\frac{1}{4\pi^{3}}\sum_{\alpha_{1}\alpha_{2}\alpha^{\prime }\alpha_{1}^{\prime }\alpha_{2}^{\prime }}\sum_{{\bar{\alpha }}_{1}{\bar{\alpha }}_{2},{\bar{\alpha }}_{1}^{\prime }{\bar{\alpha }}_{2}^{\prime }}\;\cdot$$

(35) $$ℜ\left\{W_{{\bar{\alpha }}_{2}{\bar{\alpha }}_{1}}^{*}\left({\bar{r}}^{\prime \prime },{\bar{k}}^{\prime \prime }\right)W_{\alpha^{\prime }\alpha_{1}^{\prime }}^{*}\left(r^{\prime \prime },k^{\prime \prime }\right)A_{\alpha^{\prime }{\bar{\alpha }}_{1},\alpha_{1}{\bar{\alpha }}_{1}^{\prime }}^{\mathrm{cc}*}W_{\alpha_{1}\alpha_{2}}^{}\left(r,k\right)A_{\alpha_{1}{\bar{\alpha }}_{2},\alpha_{2}^{\prime }{\bar{\alpha }}_{2}^{\prime }}^{\mathrm{cc}}W_{\alpha_{2}^{\prime }\alpha_{2}}^{*}\left(r^{\prime },k^{\prime }\right)W_{{\bar{\alpha }}_{1}^{\prime }{\bar{\alpha }}_{2}^{\prime }}^{*}\left({\bar{r}}^{\prime },{\bar{k}}^{\prime }\right)\right\}.$$

Exactly as for the density-matrix treatment previously considered, the Wigner-function version of the corresponding carrier–carrier scattering superoperator reveals again its two-body nature. Indeed, combining the general in- minus-out structure in (24) with the explicit form of the carrier–carrier scattering rates in (33) and adopting the compact notation ξ≡r,k, it is easy to realize that the carrier–carrier scattering superoperator is always of the form:

(36) $${\left.\frac{\partial f(\xi )}{\partial t}\right|}_{s}=\int d{\bar{\xi }}^{\prime \prime }d\xi^{\prime \prime }d\xi^{\prime }d{\bar{\xi }}^{\prime }\left(1-f({\bar{\xi }}^{\prime \prime })\right)\left(1-f(\xi^{\prime \prime })\right)K\left({\bar{\xi }}^{\prime \prime },\xi^{\prime \prime },\xi ,\xi^{\prime },{\bar{\xi }}^{\prime }\right)f\left(\xi^{\prime }\right)f\left({\bar{\xi }}^{\prime }\right).$$

As we can see, the scattering contribution to the Wigner function in ξ=r,k is the result of a fully nonlocal two-body transition: while the “main carrier” performs the generic transition $\xi^{\prime }=r^{\prime },$ $k^{\prime }\to \xi^{\prime \prime }=r^{\prime \prime },k^{\prime \prime }$, the “partner carrier” performs the generic transition ${\bar{\xi }}^{\prime }={\bar{r}}^{\prime },$ ${\bar{k}}^{\prime }\to {\bar{\xi }}^{\prime \prime }={\bar{r}}^{\prime \prime },{\bar{k}}^{\prime \prime }$.

## 4. Nonlocal Character of Pauli-Blocking Contributions

The aim of this section is to further investigate—both within the density-matrix formalism and within the Wigner-function picture—the role played by Pauli-blocking terms.

As discussed in [89], the time evolution of the single-particle density matrix is always characterized by a highly non-trivial coupling between diagonal (population) and non-diagonal (polarization) terms; indeed, starting from the density-matrix-based nonlinear scattering model in (15), the equation of motion for the diagonal elements $f_{\alpha }=\rho_{\alpha \alpha }$ of the semiclassical theory (see Section 2) is given by:

(37) $${\left.\frac{df_{\alpha }}{dt}\right|}_{s}=\frac{1}{2}\sum_{\alpha^{\prime }\alpha_{1}^{\prime }\alpha_{2}^{\prime }}\left(\left(\delta_{\alpha \alpha^{\prime }}-\rho_{\alpha \alpha^{\prime }}\right)P_{\alpha^{\prime }\alpha ,\alpha_{1}^{\prime }\alpha_{2}^{\prime }}^{s}\rho_{\alpha_{1}^{\prime }\alpha_{2}^{\prime }}-\left(\delta_{\alpha^{\prime }\alpha_{1}^{\prime }}-\rho_{\alpha^{\prime }\alpha_{1}^{\prime }}\right)P_{\alpha^{\prime }\alpha_{1}^{\prime },\alpha \alpha_{2}^{\prime }}^{s*}\rho_{\alpha_{2}^{\prime }\alpha }\right)\;+\;c.c.$$

This shows that the time evolution of the carrier population involves, in general, diagonal as well as non-diagonal elements; this is different from the semiclassical Boltzmann-like scattering model in (6), where all non-diagonal (polarization) terms are neglected.

In order to better compare the semiclassical scattering model in (6) with the fully quantum-mechanical result in (37), let us insert into Equation (37) the separation between population and polarization terms introduced in (1):

(38) $$\begin{array}{ccc} {\left.\frac{df_{\alpha }}{dt}\right|}_{s} & = & \sum_{\alpha^{\prime }}\left(\left(1-f_{\alpha }\right)P_{\alpha \alpha^{\prime }}^{s}f_{\alpha^{\prime }}-\left(1-f_{\alpha^{\prime }}\right)P_{\alpha^{\prime }\alpha }^{s}f_{\alpha }\right)\\ & + & \frac{1}{2}\sum_{\alpha_{1}^{\prime }\alpha_{2}^{\prime }}\left(\left(1-f_{\alpha }\right)P_{\alpha \alpha ,\alpha_{1}^{\prime }\alpha_{2}^{\prime }}^{s}p_{\alpha_{1}^{\prime }\alpha_{2}^{\prime }}-\left(1-f_{\alpha_{1}^{\prime }}\right)P_{\alpha_{1}^{\prime }\alpha_{1}^{\prime },\alpha \alpha_{2}^{\prime }}^{s*}p_{\alpha_{2}^{\prime }\alpha }\right)\;+\;c.c.\\ & - & \frac{1}{2}\sum_{\alpha^{\prime }\alpha_{1}^{\prime }}\left(p_{\alpha \alpha^{\prime }}\;P_{\alpha^{\prime }\alpha ,\alpha_{1}^{\prime }\alpha_{1}^{\prime }}^{s}\;f_{\alpha_{1}^{\prime }}-p_{\alpha^{\prime }\alpha_{1}^{\prime }}\;P_{\alpha^{\prime }\alpha_{1}^{\prime },\alpha \alpha }^{s*}\;f_{\alpha }\right)\;+\;c.c.\\ & - & \frac{1}{2}\sum_{\alpha^{\prime }\alpha_{1}^{\prime }\alpha_{2}^{\prime }}\left(p_{\alpha \alpha^{\prime }}\;P_{\alpha^{\prime }\alpha ,\alpha_{1}^{\prime }\alpha_{2}^{\prime }}^{s}\;p_{\alpha_{1}^{\prime }\alpha_{2}^{\prime }}-p_{\alpha^{\prime }\alpha_{1}^{\prime }}\;P_{\alpha^{\prime }\alpha_{1}^{\prime },\alpha \alpha_{2}^{\prime }}^{s*}\;p_{\alpha_{2}^{\prime }\alpha }\right)\;+\;c.c.\;, \end{array}$$

where $P_{\alpha \alpha^{\prime }}^{s}=P_{\alpha \alpha ,\alpha^{\prime }\alpha^{\prime }}^{s}$ denote the diagonal terms of our generalized scattering rates, which coincide with the standard semiclassical rates of the Boltzmann theory provided by the usual Fermi’s-golden-rule-prescription (see Equation (6)).

As we can see, the original scattering contribution in (37) splits into four different terms: the first one describes population–population contributions and coincides with the semiclassical model in (6), the second and third term describe, respectively, population–polarization and polarization–population contributions, while the last one describes polarization-polarization contributions, also referred to as “polarization scattering” [10]. At high carrier densities and in the presence of electronic phase coherence, these last three (polarization-induced) contributions may lead to significant modifications compared to the semiclassical case; it is however hard to draw conclusions about the impact of such quantum-mechanical corrections, since the sign of these three extra-terms depend strongly on the specific problem under investigation as well as on the device operational conditions; in contrast, in the low-density limit, the last two (polarization–population and polarization-polarization) terms vanish, and the quantum-mechanical correction with respect to the semiclassical contribution is given by the second (population–polarization) term only.

The density-matrix analysis presented so far shows that, at high carrier concentrations, the Pauli blocking factors $\left(\delta_{\alpha_{1}\alpha_{2}}-\rho_{\alpha_{1}\alpha_{2}}\right)$ may lead to significant modifications to the dissipation versus decoherence process via its diagonal (population) contributions as well as via its non-diagonal (polarization) ones.

Employing once again the Weyl–Wigner transform in (21), the above density-matrix Pauli factors are straightforwardly translated into the corresponding Pauli factors of the Wigner-function formulation (see Equations (25) and (33)):

(39) $$\sum_{\alpha_{1}\alpha_{2}}W_{\alpha_{1}\alpha_{2}}^{*}\left(r,k\right)\left(\delta_{\alpha_{1}\alpha_{2}}-\rho_{\alpha_{1}\alpha_{2}}\right)=\left(1-f(r,k)\right).$$

As shown in the previous section, within our fully quantum-mechanical Wigner-function treatment, the action of these Pauli factors is always nonlocal; this can be clearly seen in Equation (25), where the generic scattering process from $r^{\prime },k^{\prime }\to r,k$ is “weighted” by a corresponding Pauli factor $\left(1-f\left(r^{\prime \prime },k^{\prime \prime }\right)\right)$ and integrated over its phase-space coordinates $r^{\prime \prime },k^{\prime \prime }$; this implies that the impact of such nonlocal Pauli factor may be relevant, also if the value of the Wigner function in r,k is equal to zero.

**Figure 1:**
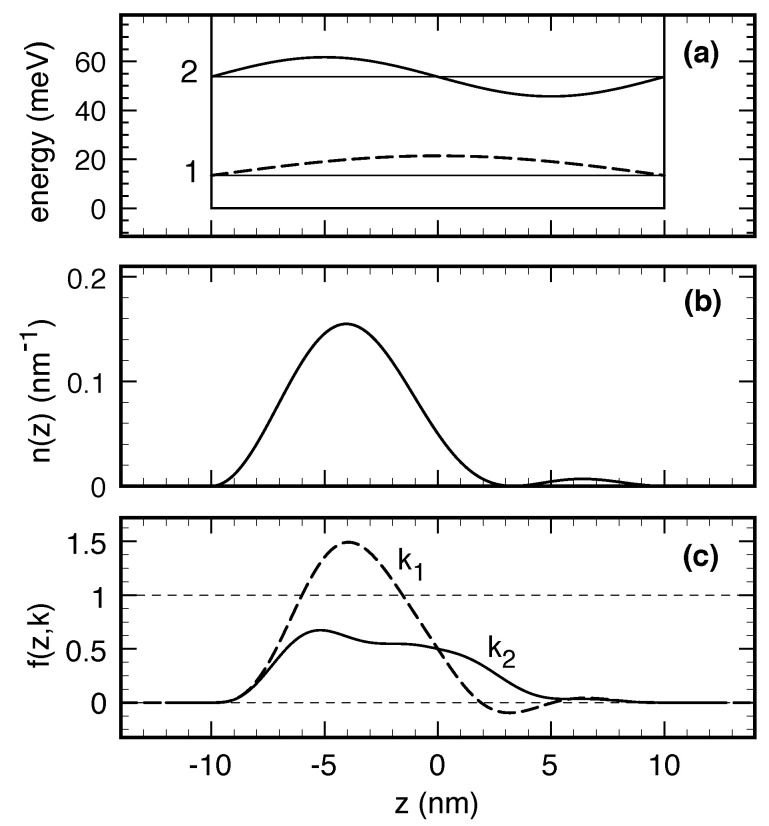
(**a**) conduction band profile along the growth (*z*) direction for the prototypical GaAs/(Al,Ga)As QW nanostructure considered in our simulated experiments. Energy levels of the first two confined states ($\epsilon_{1}$ and $\epsilon_{2}$) are shown, together with the corresponding wavefunctions ($\phi_{1}\left(z\right)$ and $\phi_{2}\left(z\right)$); (**b**) probability density ($n\left(z\right)={\left|\psi \left(z\right)\right|}^{2}$) corresponding to the coherent state in (41); (**c**) Wigner function (see Equation (43)) of the coherent state in (41) plotted for the two relevant values $k_{1}$ and $k_{2}$ corresponding to the two QW basis states in (40) (see also panel (**a**)).

We finally stress that, similar to the density-matrix case previously considered, it is difficult to evaluate the real impact of nonlocal Pauli factors within the Wigner-function picture. Indeed, as for the case of the population–polarization, polarization–population and polarization–polarization terms in (38), it is hard to draw general conclusions about the overall impact (scattering increase versus suppression) induced by such nonlocal Pauli factors. Indeed, opposite to the case of a semiclassical carrier distribution, it is imperative to recall that the Wigner function is a real quantity which may take negative values as well as values greater than one (see Figure 1c below). This implies that phase-space regions with a positive Wigner function will lead to a local suppression of dissipation versus decoherence phenomena, while phase-space regions characterized by a negative Wigner function will correspond to a Pauli factor larger than one, thus leading to a local increase of the scattering dynamics; moreover, for phase-space regions characterized by a Wigner function greater than one, the Pauli factor is negative, leading again to a scattering suppression. In a similar way, it is also important to recall that the Wigner-function scattering probabilities ${\tilde{P}}^{s,\mathrm{in}/\mathrm{out}}$ in (25) are pseudoprobabilities, i.e., real functions which, in general, are not positive-definite. This implies that, for phase-space regions where the latter are negative, the two regimes of Pauli-induced scattering suppression versus increase just discussed are simply interchanged.

As a result of the non-positive-definite character of both the Wigner function and of the corresponding scattering probabilities, we are then forced to conclude that the nonlocal Pauli blocking factors previously discussed do not necessarily lead to an overall scattering suppression; we stress that such a conclusion is in clear contrast with the behaviour predicted by semiclassical models (see Equation (6)), where the presence of local Pauli factors leads in any case to a suppression of the scattering dynamics.

In order to concretely show the intrinsic limitations of local scattering models, we shall now present a few simulated experiments of phonon-induced energy dissipation for the prototypical nanosystem depicted in Figure 1a: it consists of a l=20 nm thick GaAs quantum well (QW) surrounded by (Al,Ga)As barriers with band offset $V_{\circ }=0.3$ eV; its three-dimensional electronic states exhibit the usual subband structure due to confinement along the growth direction (*z*). To simplify our analysis, we shall neglect in-plane phase-space coordinates and adopt an effective one-dimensional (1D) description of the QW nanosystem, i.e., r,k→z,k. This implies that, within such simplified treatment, the set of single-particle quantum numbers of our nanostructure coincides with the partially discrete index of our 1D states only: α≡*n*. Moreover, since in the low-temperature simulated experiments discussed below the only electronic states involved in the dissipation process are the ground (*n* = 1) and first excited state (*n* = 2), our QW nanostructure may be described as a two-level system, whose energy levels and electronic wave functions, depicted in Figure 1a, may be safely described via the following infinite-barrier model:

(40) $$\begin{array}{ccc} k_{1}=\frac{\pi }{l}\;,\; & \epsilon_{1}=\frac{\hbar^{2}k_{1}^{2}}{2m^{*}}\;, & \;\phi_{1}\left(z\right)=\sqrt{\frac{2}{l}}\;\cos\left(k_{1}z\right),\\ k_{2}=\frac{2\pi }{l}\;,\; & \epsilon_{2}=\frac{\hbar^{2}k_{2}^{2}}{2m^{*}}\;, & \;\phi_{2}\left(z\right)=-\sqrt{\frac{2}{l}}\;\sin\left(k_{2}z\right) \end{array}$$

($m^{*}$ denoting the GaAs effective mass). The prototypical QW nanostructure in Figure 1a has been optimized in order to maximize the intersubband carrier–phonon coupling; indeed, for l=20 nm, the interlevel splitting ($\epsilon_{2}-\epsilon_{1}≃40$ meV) matches with the GaAs LO-phonon energy [6].

In order to better emphasize the intrinsic limitations of local scattering models, let us consider an electronic state given by a coherent and equally weighted superposition [94] of the two QW basis states in (40), namely

(41) $$\psi \left(z\right)=c_{1}\phi_{1}\left(z\right)+c_{2}\phi_{2}\left(z\right)\;,\;c_{1}=c_{2}=\frac{1}{\sqrt{2}}\;,$$

whose probability density $n\left(z\right)={\left|\psi \left(z\right)\right|}^{2}$ is depicted in Figure 1b. It is easy to show that the coherent electronic state in (41) corresponds to the following (two-by-two) single-particle density matrix [95]:

(42) $$\left(\begin{array}{cc} \rho_{11} & \rho_{12}\\ \rho_{21} & \rho_{22} \end{array}\right)=\left(\begin{array}{cc} {\left|c_{1}\right|}^{2} & c_{1}^{}c_{2}^{*}\\ c_{2}^{}c_{1}^{*} & {\left|c_{2}\right|}^{2} \end{array}\right)=\left(\begin{array}{cc} \frac{1}{2} & \frac{1}{2}\\ \frac{1}{2} & \frac{1}{2} \end{array}\right).\;$$

As for any pure state ψ(z), the corresponding Wigner function is simply given by:

(43) $$f\left(z,k\right)=\int dz^{\prime }\psi^{}\left(z+\frac{z^{\prime }}{2}\right)e^{-ıkz^{\prime }}\psi^{*}\left(z-\frac{z^{\prime }}{2}\right).\;$$

Figure 1c shows the above Wigner function for the two relevant values $k_{1}$ and $k_{2}$ corresponding to the two QW basis states in (40) (see also Figure 1a). In addition to the strongly asymmetric nature of both the probability density n(z) in Figure 1b and of the corresponding Wigner function profiles in Figure 1c, the latter exhibit negative values as well as values significantly greater than one (see dashed curve).

Combining Equations (24) and (25), the 1D version (r,k→z,k) of the nonlocal scattering model in (24) for the case of carrier–phonon interaction comes out to be:

(44) $${\left.\frac{\partial f(z,k)}{\partial t}\right|}_{s}=\int \frac{dz^{\prime \prime }dk^{\prime \prime }\;dz^{\prime }dk^{\prime }}{2\pi }\left(1-f(z^{\prime \prime },k^{\prime \prime })\right)\Delta {\tilde{P}}^{\mathrm{cp}}\left(z^{\prime \prime },k^{\prime \prime };z,k;z^{\prime },k^{\prime }\right)f\left(z^{\prime },k^{\prime }\right)$$

with

(45) $$\Delta {\tilde{P}}^{\mathrm{cp}}\left(z^{\prime \prime },k^{\prime \prime };z,k;z^{\prime },k^{\prime }\right)={\tilde{P}}^{\mathrm{cp},\mathrm{in}}\left(z^{\prime \prime },k^{\prime \prime };z,k;z^{\prime },k^{\prime }\right)-{\tilde{P}}^{\mathrm{cp},\mathrm{out}}\left(z^{\prime \prime },k^{\prime \prime };z,k;z^{\prime },k^{\prime }\right)\;.$$

Here, ${\tilde{P}}^{\mathrm{cp},\mathrm{in}/\mathrm{out}}$ are the 1D version of the fully nonlocal scattering rates in (30)–(31), and, for the case of our simplified QW model, the generalized carrier–phonon scattering rates in (16) acquire the diagonal form: $P_{\alpha_{1}\alpha_{2},\alpha_{1}^{\prime }\alpha_{2}^{\prime }}^{\mathrm{cp}}=P_{\alpha_{1}\alpha_{1}^{\prime }}\delta_{\alpha_{1}\alpha_{1}^{\prime },\alpha_{2}\alpha_{2}^{\prime }}$. In particular, in the low-temperature limit, the only active relaxation channel is the 2→1 transition induced by LO-phonon emission, namely

(46) $$\left(\begin{array}{cc} P_{11} & P_{12}\\ P_{21} & P_{22} \end{array}\right)=\left(\begin{array}{cc} 0 & P^{\circ }\\ 0 & 0 \end{array}\right),\;$$

where for our GaAs-based QW nanostructure the 2→1 phonon-emission rate $P^{\circ }$ is of the order of 5 ps${}^{-1}$.

In order to compare the fully nonlocal QW scattering model described so far with its local counterpart, we shall describe energy relaxation via an effective Boltzmann-like equation coupling the two energy levels of the QW nanostructure depicted in Figure 1a. According to such a local scattering model, the phonon-induced time evolution of the upper-level Wigner function (see solid curve in Figure 1c) is given by:

(47) $${\left.\frac{\partial f(z,k_{2})}{\partial t}\right|}_{s}=\left(1-f(z,k_{2}\right)P_{21}f\left(z,k_{1}\right)-\left(1-f(z,k_{1}\right)P_{12}f\left(z,k_{2}\right)\;,$$

and in the low-temperature limit (see Equation (46)), the latter reduces to:

(48) $${\left.\frac{\partial f(z,k_{2})}{\partial t}\right|}_{s}=-\;\left(1-f(z,k_{1}\right)P^{\circ }f\left(z,k_{2}\right)\;.$$

**Figure 2:**
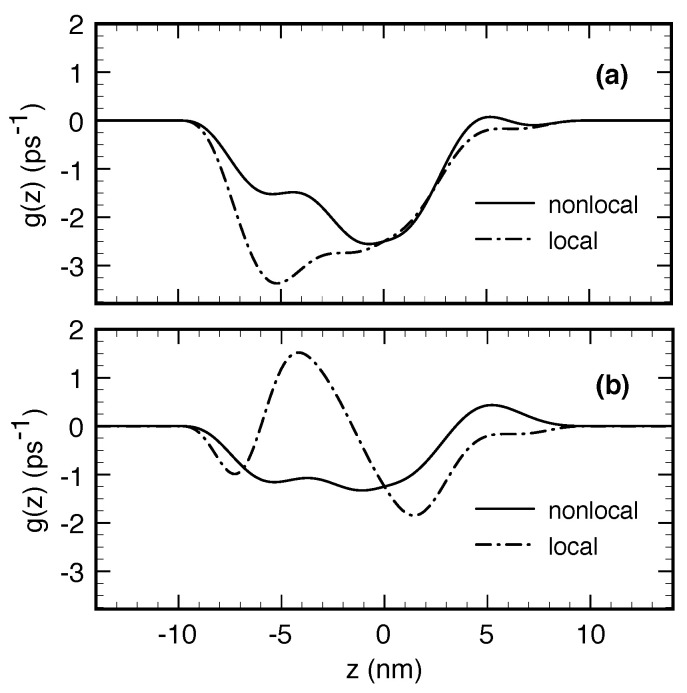
Time derivative of the upper-level Wigner-function profile (see Equation (49)): comparison between the nonlocal model in (44) (solid curves) and its local counterpart in (47) (dash-dotted curves) in the absence (**a**) and presence (**b**) of Pauli-blocking terms (see text).

In Figure 2, we show the time derivative

(49) $$g\left(z\right)={\left.\frac{\partial f(z,k_{2})}{\partial t}\right|}_{s}$$

of the upper-level Wigner-function profile (see solid curve in Figure 1c) comparing the nonlocal model in (44) (solid curves) with its local counterpart in (47) (dash-dotted curves) in the absence (a) and presence (b) of Pauli-blocking terms.

As we can see, already neglecting Pauli-blocking factors, the nonlocal and local scattering models exhibit qualitatively different behaviours. Indeed, while the local result (dash-dotted curve in (a)) is always negative and simply proportional to the Wigner function $f(z,k_{2})$ (see Equation (48) and solid curve in Figure 1c), the nonlocal one (solid curve in (a)) comes out to be significantly different. This is due to the nonlocal nature of the carrier–phonon scattering model in (44) present also in the absence of the Pauli factor $\left(1-f(z^{\prime \prime },k^{\prime \prime })\right)$ and ascribed to the spatial integration with respect to $z^{\prime }$.

In the presence of Pauli-blocking factors, the discrepancies between nonlocal and local models are strongly amplified. Indeed, while for the nonlocal model the presence of the Pauli factors leads basically to an overall suppression of the time derivative (solid curve in (b)), with respect to the Pauli-free case (solid curve in (a)), the local result (dash-dotted curve in (b)) exhibits significant positive-definite regions, due to negative values of the Pauli factor $\left(1-f(z,k_{1})\right)$.

As a confirmation of the intrinsic limitations of the local scattering model pointed out so far, it is easy to show that the Wigner function of the QW ground state $\phi_{1}\left(z\right)$—corresponding to the zero-temperature equilibrium state of our nanostructure—is not a steady-state solution of the local scattering model in (48).

## 5. Conclusions

Thanks to their simple physical interpretation as well as to their straightforward implementation within various quantum-mechanical simulation schemes, semiclassical scattering models have been widely employed in the design and optimization of new-generation quantum nanomaterials and related nanodevices. In particular, during the last few decades, two different classes of semiclassical treatments have been independently used, namely density-matrix and Wigner-function schemes. The first class is based on the so-called diagonal approximation, i.e., the neglect of non-diagonal density-matrix elements (i.e., polarization terms). The second class includes local scattering models borrowed from the conventional Boltzmann transport theory, namely relaxation-time schemes as well as Boltzmann collision terms; it has been recently shown [79] that the use of such local scattering approaches within the Wigner-function formalism may lead to unphysical results, namely anomalous suppression of intersubband relaxation, incorrect thermalization dynamics, and violation of probability-density positivity. To overcome such severe limitations, a quantum-mechanical generalization of relaxation-time and Boltzmann-like models has been recently proposed [79], resulting in nonlocal electron-phonon scattering superoperators.

The primary goal of this paper is twofold: on the one hand, we have investigated the intimate link between density-matrix and Wigner-function approaches, pointing out intrinsic limitations of semiclassical scattering models within these, apparently different, simulation strategies. On the other hand, we have extended the carrier–phonon treatment in [79] to carrier–carrier interaction, deriving the explicit form of the corresponding two-body scattering superoperator.

The main result of our investigation is that, for both carrier–phonon and carrier–carrier interaction, it is hard to evaluate the impact (scattering suppression or increase) of Pauli-blocking factors. More specifically, within the density-matrix picture, such terms give rise to quantum corrections (with respect to the semiclassical case), namely population–polarization, polarization–population, and polarization-polarization terms, often referred to as “polarization scattering”. At the same time, within the Wigner-function picture, the action of the corresponding Pauli factors comes out to always be nonlocal. Combining such nonlocal character with the non-positive-definite nature of both the Wigner function and of the corresponding scattering probabilities, it is again hard to draw general conclusions on the overall impact of Pauli blocking terms on energy dissipation and decoherence processes.

In order to concretely show the intrinsic limitations of local scattering models, a few simulated experiments of energy dissipation and decoherence in a QW semiconductor nanostructure have also been presented. The latter show that, already in the low-density limit (i.e., neglecting Pauli-blocking terms), one deals with significant nonlocal corrections, and that, at high carrier densities, these corrections are strongly amplified.
